# A Reliable Way to Detect Endogenous Murine β-Amyloid

**DOI:** 10.1371/journal.pone.0055647

**Published:** 2013-02-01

**Authors:** Andrew F. Teich, Mitesh Patel, Ottavio Arancio

**Affiliations:** Department of Pathology and Cell Biology, Taub Institute for Research on Alzheimer's Disease and the Aging Brain, Columbia University, New York, New York, United States of America; University of North Dakota, United States of America

## Abstract

Unraveling the normal physiologic role of β-amyloid is likely crucial to understanding the pathogenesis of Alzheimer's disease. However, progress on this question is currently limited by the high background of many ELISAs for murine β-amyloid. Here, we examine the background signal of several murine β-amyloid ELISAs, and conclude that the majority of the background is from non-APP derived proteins. Most importantly, we identify ELISAs that eliminate this background signal.

## Introduction

The Amyloid Hypothesis of Alzheimer's disease states that the accumulation of high levels of cerebral β-amyloid protein is a central event in this illness [1]. This hypothesis has subsequently fueled a massive research effort into understanding the physiology of β-amyloid protein. Although β-amyloid may reach high (nanomolar) concentrations in Alzheimer's disease [2], β-amyloid is also produced in the brain throughout life, and the normal *in vivo* concentration in the rodent brain has been estimated to be in the picomolar range [3], [4]. Understanding how β-amyloid is normally regulated may help us understand how it accumulates to high levels in Alzheimer's disease, and this realization has inspired investigation into the normal physiologic function of β-amyloid [4], [5], [6], [7], [8]. In addition, others have recently started using wild-type rodents to study the function of γ-secretase inhibitors [9], [10]. In all of these cases, studying β-amyloid in wild-type animals requires a reliable method of measuring low, picomolar concentrations of endogenous cerebral β-amyloid. However, it has been noted by several groups that ELISAs that use common β-amyloid antibodies (such as 4G8 and Signet 9153) give a very high background reading with wild-type rodent brain, presumably due to non-specific binding of various rodent brain proteins with these antibodies [10], [11], [12]. This has prompted some authors to claim that the ELISA method is flawed at measuring endogenous murine β-amyloid [11], [12]. To correct this flaw, a solid-phase extraction technique has been proposed to chromatographically separate wild-type rodent β-amyloid from the proteins in rodent brain that lead to this non-specific binding [11], [12]. However, some investigators who wish to measure endogenous murine β-amyloid may not have expertise with solid-phase extraction, which is a technique that is not commonly used in the Alzheimer's disease scientific community. In this study, we use hippocampal tissue from amyloid precursor protein knock-out (APP-KO) mice and wild-type (WT) littermates to investigate this issue further. We conclude that 1) The majority of the background signal seen in ELISAs for murine β-amyloid is from non-APP derived proteins, and most importantly, 2) We identify ELISAs that eliminate this background signal.

## Materials and Methods

### Animals

APP-KO mice [13] were bread against a B6 background; all mice were purchased from Jackson labs. Hemizygous transgenic (Hu*APP*695SWE)2576 mice expressing mutant human *APP* (K670N,M671L) [14] were used as positive controls in several experiments (these mice are from a colony that derives from a gift from Karen Hsiao-Ashe). Unless otherwise specified, mice were between 4 and 8 months (adults) when they were sacrificed for tissue analysis. This study was carried out in strict accordance with the recommendations in the Guide for the Care and Use of Laboratory Animals of the National Institutes of Health. The protocol was approved by the Institutional Animal Care and Use Committee of Columbia University (Protocol Number: AC-AAAD9255).

### ELISA

Mouse hippocampi were homogenized in 880 µl of tissue lysate buffer (20 mM Tris-HCl (pH 7.4), 1 mM ethylenediaminetetraacetic acid, 1 mM ethyleneglycoltetraacetic acid, 250 mM sucrose) supplemented with protease inhibitors (Roche). The tissue homogenates were treated with diethanolamine to extract soluble β-amyloid. We did not perfuse the mice with heparin before-hand, as our goal is to compare the ability of various ELISAs to measure β-amyloid without this requirement. Although heparin perfusion is sometimes done to eliminate IgG from the brain, it is difficult to do this when measuring acute changes in β-amyloid, or when measuring β-amyloid in a hippocampal slice preparation. Thus, we sought to determine whether there is an ELISA technique that can measure β-amyloid in all circumstances without any requirements for prior heparin perfusion.

We measured β-amyloid in our hippocampal homogenate using the following ELISA kits: Covance Colorimetric BetaMark™ Beta-Amyloid x-42 ELISA Kit (Catalog Number: SIG-38956), Covance Colorimetric BetaMark™ Beta-Amyloid x-40 ELISA Kit, (Catalog Number: SIG-38954), Invitrogen Aβ 42 Mouse ELISA Kit (Catalog Number: KMB3441), Invitrogen Aβ 40 Mouse ELISA Kit (Catalog Number: KMB3481), Wako Human/Rat(Mouse) β-Amyloid (40) ELISA Kit (Catalog Number: 294-62501), and Wako Human/Rat(Mouse) β-Amyloid (42) ELISA High-Sensitive Kit (Catalog Number: 292-64501). All ELISA assays were performed according to the manufacturer's protocol. In addition, ELISAs using either 6E10 or M3.2 as a capture antibody and an HRP-conjugated 4G8 as a detection antibody were prepared as previously described [11] (all antibodies were purchased from Covance). Costar 96-well plates were incubated overnight at 4°C with capture antibody (in 0.1 M sodium bicarbonate, pH 8.2) at a dilution of 4 µg/mL. Plates were blocked the following morning in Block Ace (AbD Serotech) and then incubated overnight with 50 µl of brain lysate. The following morning the plates were washed with PBST and then incubated for 2 hours at room temperature with HRP-4G8 (Covance) at a dilution of 1 µg/mL. Plates were then washed and read at 620 nM wavelength 30 minutes after adding 100 µl of TMB substrate (Covance). The signal was normalized to the protein concentration for each sample. Between 5 and 6 mice were used in each group when comparing WT and APP-KO tissue for a given ELISA. All data analysis was done using Microsoft Excel; statistical significance was calculated using a 2-tailed T-test.

### Western Blot

Western blotting was performed as previously described [15]. Hippocampal tissue was homogenized in RIPA buffer (Fisher scientific) with protease inhibitor (Roche) at 4°C, followed by centrifugation at 2,000 rpm for 1 min. The supernatant was electrophoresed on 4–12% Bis-Tris NuPAGE gels (Invitrogen) and then immunoblotted on nitrocellulose membrane. All primary antibodies were used at a 1∶1,000 concentration for immunoblotting. HRP-conjugated secondary antibodies were purchased from Millipore. Although we do not quantify the bands in this paper, the same amount of protein (30 µg) was loaded for each sample for ease of comparison.

## Results

**Figure 1:**
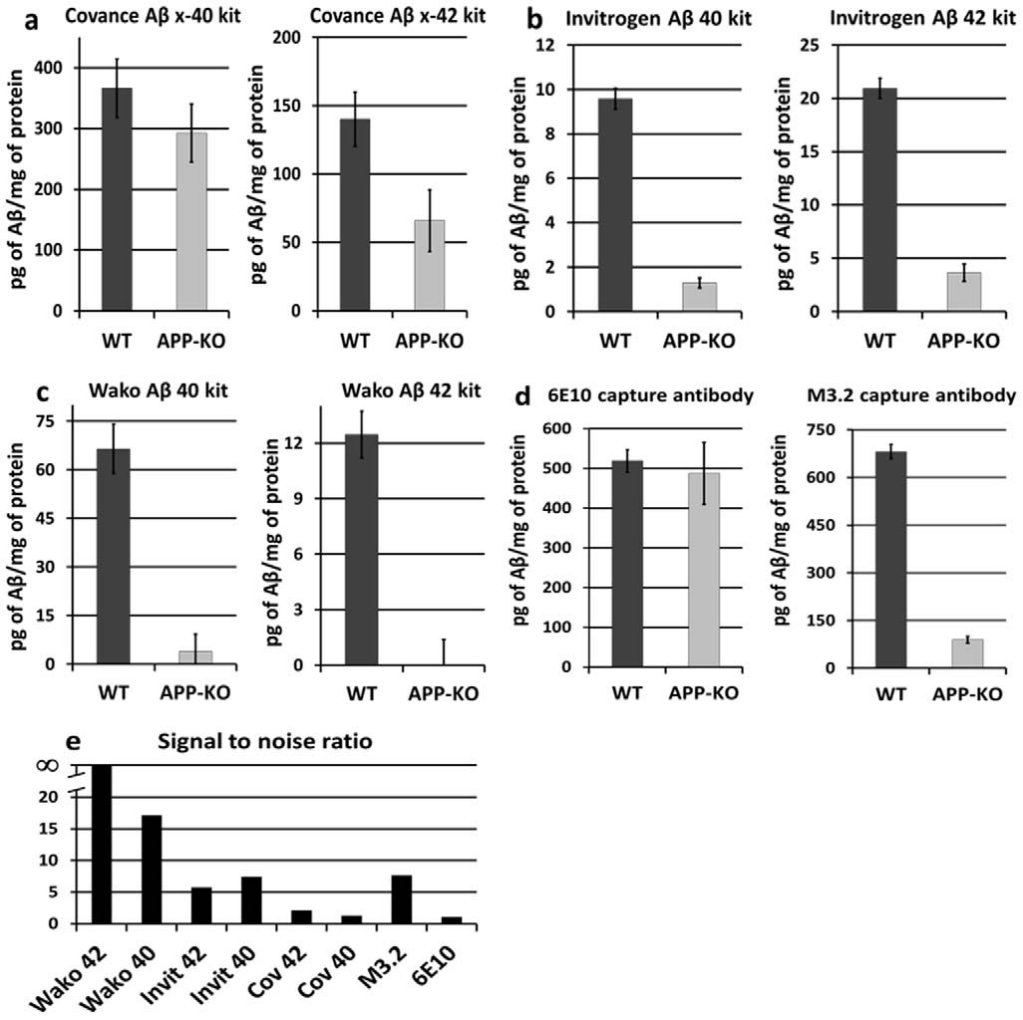
The amount of background signal with APP-KO tissue varies widely between different ELISAs. **A**) The Covance Colorimetric BetaMark™ Beta-Amyloid x-40 ELISA Kit (left) and x-42 Kit (right). Both kits give a signal with APP-KO tissue that is significantly above baseline. The x-40 kit gives a signal with APP-KO tissue that is not significantly different from WT (the difference between APP-KO and WT is significant for the x-42 kit). **B**) The Invitrogen Aβ 40 Mouse ELISA Kit (left) and Aβ 42 Mouse ELISA Kit (right). Both kits give a signal with APP-KO tissue that is significantly above baseline, but also significantly different from WT tissue. **C**) The Wako β-Amyloid (40) ELISA Kit (left) and β-Amyloid (42) ELISA High-Sensitive Kit (right). Both kits give a signal with APP-KO tissue that is not statistically significant from baseline. **D**) Both WT and APP-KO give a similar level of background signal with an ELISA for human β-amyloid (6E10 capture antibody – left), but show a clear difference when a rodent-specific capture antibody is used (M3.2 antibody – right). **E**) “Signal to noise” ratio of the WT signal divided by the APP-KO signal for each ELISA. Error bars in A–D are standard error.

When interpreting the signal from a β-amyloid ELISA, we assume that any signal with APP-KO tissue is pure noise, whereas signal from WT tissue is noise plus genuine signal from murine β-amyloid. We first compared the ability of six commercially available ELISAs from three different companies to distinguish WT from APP-KO hippocampal lysate (see Methods for details and catalog numbers). We first tried the Covance β-amyloid x-40 and x-42 ELISA kits. We found that both kits gave a strong signal for both WT and APP-KO mice (Figure 1A). In fact, there was no significant difference between WT and APP-KO mice for the x-40 kit (p-value = 0.3). For the x-42 kit the difference was significant (p-value = 0.03), but there was still a strong signal generated by APP-KO hippocampal tissue. For both of the Covance kits, the signal generated by the APP-KO tissue was statistically different from blank (p-value = 0.0001 for the x-40 kit; p-value = 0.015 for the x-42 kit). We next tried the Invitrogen mouse β-amyloid 40 and β-amyloid 42 kits. Both kits gave a substantial and statistically significant difference between WT and APP-KO mice (Figure 1B). However, the APP-KO signal was still statistically different from blank in both cases (p-value = 0.002 for the β-amyloid 42 kit; p-value = 0.0004 for the β-amyloid 40 kit). Interestingly, the total amount of β-amyloid detected by the Invitrogen kits is significantly less than the amount detected by the Covance kits (when normalized by protein levels). This may be due to the fact that the Covance kits are detecting significant amounts of other proteins in addition to β-amyloid. Consistent with this second possibility, the APP-KO tissue has a strong signal with the Covance kits. Finally, we ran the same experiment with the β-Amyloid 40 and β-Amyloid 42 High-Sensitive kits from Wako chemicals. These kits both gave a large, statistically significant difference between WT and APP-KO tissue (Figure 1C). In fact, the β-Amyloid 42 High-Sensitive kit gave a signal with the APP-KO tissue that was effectively at our blank reading (it was actually slightly below). The Wako β-Amyloid 40 kit gave a positive background reading with APP-KO tissue, but it was not statistically significantly different from the blank reading (p-value = 0.49).

How much of the background noise in the above ELISAs is from fragments of APP other than β-amyloid and how much is from non-APP derived proteins? Since APP-KO mice give a robust signal with many of the above kits, we assume that a large portion of the noise in these kits is due to proteins not derived from APP. However, there remains the possibility that some of the signal in WT mice is coming from APP-derived fragments other than β-amyloid. Definitively answering this question is somewhat tricky, as APP has multiple proteolytic fragments. In addition, most commercially available ELISA kits do not disclose which antibodies are used in them; this is true both for the Covance and the Invitrogen kits that had significant background activity from APP-KO tissue. Thus, in order to further investigate this issue, we decided to design an ELISA that was specific for human β-amyloid, and measure the signal from WT and APP-KO tissue with this ELISA. This ELISA should not detect murine β-amyloid [11]. Thus, if the signal from APP-KO and WT mice is equal in this ELISA, then this argues that at least in this case, the noise is coming entirely from non-APP derived proteins. To do this, we made an ELISA using 6E10 (a human-specific β-amyloid antibody) as the capture antibody and HRP-conjugated 4G8 (which recognizes both rodent and human β-amyloid) as the detection antibody. As seen in Figure 1D, both APP-KO and WT mice gave a strong signal with this ELISA, and both signals are of comparable strength. To verify that our ELISA was working correctly, we first successfully measured step-wise dilutions of human synthetic β-amyloid (data not shown). As an additional control, we then asked whether this ELISA could have detected murine β-amyloid if the capture antibody had been for rodent β-amyloid. To do this, we ran the exact same ELISA with APP-KO and WT tissue, but changed the capture antibody from human-specific 6E10 to rodent-specific M3.2; the ELISA was identical in all other respects. This ELISA should now give a clear difference between WT and APP-KO tissue, although there may well be some background signal with the APP-KO tissue. As expected, this ELISA gave a strong signal in WT tissue and a smaller, but statistically significant signal in APP-KO tissue (Figure 1D). The very strong signal seen with the WT tissue in the M3.2 ELISA suggests that there is a significant contribution from proteins other than β-amyloid. Since the APP-KO tissue only gives a small signal, it is likely that this ELISA not only cross-reacts with other non-APP related proteins, but also cross-reacts with other proteins derived from APP other than β-amyloid. The M3.2 and 4G8 antibodies are known to cross-react with multiple APP fragments [16], [17]. Thus, it is reasonable to assume that other APP-related fragments partially contribute to the WT signal in our M3.2 ELISA.

We next quantified the “signal to noise” ratio for each ELISA we had run (Figure 1E), which we define as the ratio of the WT signal to APP-KO signal for each ELISA. Note that we give the Wako β-Amyloid 42 High-Sensitive kit a ratio of “∞” because the denominator is zero. Also note that the Invitrogen kits and the M3.2 homemade ELISA all give a large difference between WT and APP-KO brain tissue. However, because the APP-KO signal is statistically significant in all of these ELISAs, the signal/noise ratio for these ELISAs is much smaller than the Wako ELISA kits.

**Figure 2:**
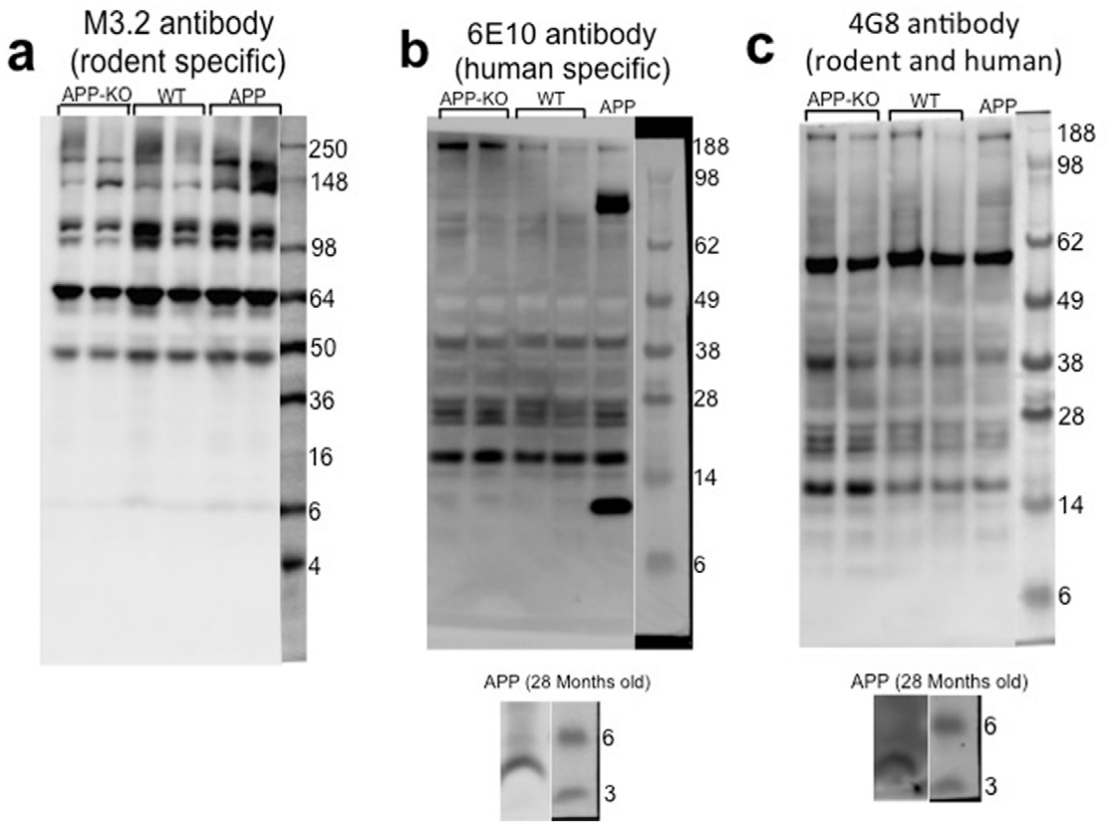
Several β-amyloid antibodies show high non-specificity for β-amyloid by western blot. **A**) Western blot with the antibody M3.2 (specific for rodent β-amyloid) using hippocampal tissue from APP-KO, WT, and APP transgenic mice expressing human APP (Hu*APP*695SWE). All three genotypes show a similar band pattern. **B**) Western blot with the antibody 6E10 (specific for human β-amyloid). All three genotypes show a similar band pattern. However, with 6E10, the APP transgenic mouse tissue shows an additional 8 kD band (presumably a human β-amyloid dimer) as well as a band near 87 kD (presumably full-length human APP). In addition, a 4.5 kDa band (β-amyloid) is detected when older mouse brain is used that has high levels of β-amyloid protein. **C**) Western blot with 4G8. All three genotypes also showed a similar band pattern with this antibody as well. In addition, a 4.5 kDa band (β-amyloid) is detected when older mouse brain is used that has high levels of β-amyloid protein.

Finally, we ran western blots using the three antibodies from our homemade ELISAs (M3.2, 4G8, and 6E10) on hippocampal tissue from WT, APP-KO, and APP transgenic mice expressing human APP (Hu*APP*695SWE) (Figure 2). Although antibodies may bind proteins in a western blot that they do not bind in an ELISA [2], we did this experiment as an additional test of antibody specificity for β-amyloid. Surprisingly, WT and APP-KO tissue showed a very similar band pattern for all three antibodies. 4–8 month old APP transgenic tissue also showed a similar band pattern, although 4G8 and 6E10 detected β-amyloid when brain tissue from 28 month old transgenic mice was used. In addition, 6E10 detected an 87 kD band in APP transgenic tissue that is probably full-length human APP. In summary, the similar band pattern between WT and APP-KO tissue is consistent with our general conclusion that many β-amyloid antibodies show non-specific binding. Because we have used APP-KO tissue in this analysis, this also supports the conclusion that much of the non-specific binding is with proteins that are not derived from APP.

## Discussion

In conclusion, we have confirmed prior reports of non-specificity of β-amyloid antibodies when performing ELISAs for murine β-amyloid with rodent brain tissue [10], [11], [12]. By performing our experiments with APP-KO mice, we have demonstrated that much of the observed cross-reactivity is not with other proteolytic fragments of APP, but with other non-APP related proteins found in rodent brain. Most importantly, we have shown that not all β-amyloid ELISAs show high non-specificity, and investigators may detect endogenous murine β-amyloid in rodent brain tissue using an ELISA without a prior solid-phase extraction step. Finally, we want to emphasize that our findings here pertain to the detection of *endogenous* murine β-amyloid. For example, the Covance kits will have less of an issue measuring fluctuations of human β-amyloid in transgenic animals. This is because the huge amount of β-amyloid that is produced in transgenic mice will be much higher than the background signal. For example, some authors have estimated that 35 times as much β-amyloid is being produced in transgenic mice than in wild-type rodents [10]. Thus, the Covance kits may still be quite useful in these kinds of experiments.

Measuring natural variations in murine endogenous β-amyloid is important for understanding the physiology of this protein, and the work done here will hopefully make it easier for researchers to select the appropriate kit for their work. An interesting side issue is whether there are significant differences in endogenous β-amyloid levels in different strains of mice, and whether this affects the choice of kit for different mouse strains. In our own work we have used the Wako high-sensitive β-amyloid 42 kit on several different strains of mice (C57B6, FVB, C57B6/129 hybrids, and C57B6/Swiss Webster hybrids), and we have found roughly comparable levels of β-amyloid 42. Thus, although the work in this paper was done with C57B6 mice, we believe that the results extrapolate to other mouse strains as well.

In table 1, we summarize the strengths and weaknesses of the six commercially available kits tested here. In summary, the ELISAs tested in this paper range from being rodent-specific (Invitrogen) to working with both rodents and humans (Covance and Wako). The fact that the Invitrogen kit is rodent specific gives this kit a unique advantage over the Covance and Wako kits. Namely, the Invitrogen kit can be used to measure the physiology of endogenous rodent β-amyloid in transgenic mice that also express human β-amyloid. Any experiment that examines endogenous β-amyloid processing in transgenic mice must use this kit, as the Wako and Covance kits will not distinguish between endogenous and human β-amyloid. The signal-to-noise ratio ranges from poor (Covance) to excellent (Wako). Thus, in terms of signal-to-noise ratio, the Wako kits are the best. Finally, cost must be a consideration when choosing an ELISA kit. The Covance kits are cheaper than either the Invitrogen or Wako kits, so this may be a reason to choose these kits if they fit the experiment one is trying to do (for example, measure human β-amyloid in a transgenic mouse).

**Table 1:** Summary of Commercially Available Kits We Tested.

	Covance Aβ x-40	Covance Aβ x-42	Invitrogen Aβ 40	Invitrogen Aβ 42	Wako Aβ 40	Wako Aβ 42
**Species**	Rodent and Human	Rodent and Human	Rodent	Rodent	Rodent and Human	Rodent and Human
**Signal to Noise Ratio**	Poor	Poor	Good	Good	Excellent	Excellent
**Cost**	Lowest	Lowest	Low-Intermediate	Low-Intermediate	Highest	Highest
